# Development of the Japanese Parenting Style Scale and examination of its validity and reliability

**DOI:** 10.1038/s41598-022-23153-5

**Published:** 2022-10-27

**Authors:** Keisuke Okubo, Yinqi Tang, Jiwon Lee, Toshihiko Endo, Sachiko Nozawa

**Affiliations:** 1 The Center for Early Childhood Development, Education, and Policy Research, The University of Tokyo, Tokyo, Japan; 2 Kyoai Gakuen University, Maebashi, Japan; 3 Benesse Educational Research and Development Institute, Tokyo, Japan

**Keywords:** Psychology, Human behaviour

## Abstract

Parenting is an essential factor affecting child development. Therefore, several studies have focused on individual differences in parenting (i.e., parenting styles). However, there exist only a few useful scales in Japan, especially for parents who have preschool children. Therefore, a new scale for assessing parenting styles in Japan, based on the traditional theoretical framework, was developed, and examined for its validity and reliability. In Study 1, 82 original items were constructed and 1236 parents with preschool children completed these items. Next, 28 items for the Japanese Parenting Style Scale (JPSS) were selected based on factor analysis and the analyses of the graded response model. The JPSS included four factors: warmth, hostility, permissiveness, and harsh control. The results showed that each sub-scale had sufficient conceptual validity and internal consistency. In Study 2, the criterion-related validity of the JPSS was examined. A total of 1236 parents, non-participants in Study 1, completed the JPSS and other scales. The results showed sufficient criterion-related validity for the scale.

## Introduction

Parenting is an essential factor influencing children’s development^1,2^. Since Baldwin^3^ revealed parenting characteristics affecting children’s socialization, numerous studies have focused on the individual differences in parenting (i.e., parenting styles) over the past 75 years. In general, parenting styles refer to emotional climate parents raise their children in^4^, whereas parenting practices refer to the specific behaviors used by parents to socialize with their children, such as helping them with homework and being involved in their schooling. In the present study, parenting styles and practices were distinguished based on the suggestions of Darling and Steinberg^4^ and this study focused on parenting styles since several studies have shown that they determine the quality of parenting practices^5^. Previous studies have shown that parenting style is associated with several child development aspects, including socio-emotional skills, cognitive ability, and lifelong health^6–10^. Therefore, several studies have attempted to categorize parenting styles in various ways.

Research regarding parenting styles started with Lewin’s study on leadership style in adults^11,12^. They classified leadership styles into three categories (democracy, authoritarian, and laissez-faire). Baldwin^13^ recognized the importance of Lewin’s study and applied this classification to the understanding of parenting styles. He divided democracy into two styles; scientific democracy and warm democracy^13,14^. Of those two styles, warm democracy, which is characterized by behavior that provides a great deal of freedom to the child, was associated with positive child development; however, also with antisocial behavior. The results indicated that indulgent parenting may lead to an increase in externalizing problems in children. Accordingly, Baumrind focused on controlling parenting, and discriminated between positive and negative control. He captured parenting styles using three well-known classifications: authoritative, authoritarian, and permissive^15,16^. While authoritative style is characterized by control with warmth or responsiveness, authoritarian style is characterized by control with cold and non-responsive attitudes. About 10 years after Baumrind’s typological classification, Maccoby and Martin^17^ captured the parenting style in two dimensions: controling and responsive (i.e., warmth). Three of the four parenting styles classified in that two dimensions correspond to the three categories classified by Baumrind. The fourth parenting style, characterized by less controling and responsive was labeled as neglectful parenting. Further, a recent meta-analysis on the components of parenting styles identified that they can be grouped into four clusters: positive, controlling, harshness, and uninvolved^18^. There are some other factors that are identified as components of parenting style, but basically, parenting style has been captured by the factors described in this section.

There are two peer-reviewed scales in Japan that measure parenting styles, including translated versions of scales developed in Western countries. However, they have some problems. First, the items developed in other cultures do not correspond with the Japanese culture; therefore, using the translated items would distort the participants’ responses. For example, the Japanese version of the Parenting Scale^19^ is a translated version of the Parenting Scale^20^ which consists of two factors: over-reactivity and laxness^21^. As this scale focuses on the negative aspects of parenting, it is useful for assessing a clinical sample. Nevertheless, a few items in this scale are difficult for Japanese people to answer since the scale is composed of only the translated items. As a result, the distribution of responses to these items was skewed.

Another difficulty is the absence of a scale that can measure the classical classification of parenting styles as described in the previous section. Although there is a scale composed of newly created Japanese items that aimed to overcome the cultural incompatibility of the translated items (the Positive and Negative Parenting Scale^22^), this scale were not based on the theoretical background of parenting styles, as described above. Another problem with the scale is that they often contain items that are too specific (i.e., items that measure specific parenting practices rather than the parenting climate). In summary, the scales currently available in Japanese to measure parenting styles have problems in that the content of the items is not suitable for Japanese parents and that they disregard the theoretical background, and it is necessary to develop a scale that can address these problems.

Accordingly, the purpose of this study is to develop a scale consisting of items that are easily understandable for Japanese parents on the basis of theoretical background. The present study focuses on parenting preschool children because the existing scales mainly focused on parenting school age children^22^. The importance of parenting as a determinant of child development is relatively large in early childhood, when the influence of other interpersonal relationships (i.e., friendships) is less during the school age^4^. In fact, parenting styles are one of the most important factors that determine various aspects of development of preschool children^23–25^. Since there is no useful scale in Japan to measure the parenting styles of parents with preschool children, development of the scale would advance research and practice targeting them. The present study did not focused on parents of early infants because caregiving plays an important role in parenting behaviors during infancy different from latter developmental stages^26^. Research on parenting in infancy primarily assessess specific aspects of caregiving, such as sensitivity^27^, by using behavioral indices instead of capturing individual differences of parenting styles. For this reason, parents of early infants were excluded from the interest in this study.

In summary, the present study aims to develop a new scale for assessing parenting styles with new items. In Study 1, an item pool was developed based on theoretical assumptions and appropriate items for the scale were selected. Next, the validity of the factor structure and reliability of measurement for each sub-scale was examined. In Study 2, validity of the constructed scale in terms of concurrent correlations with other scales was further examined.

## Study 1

### Methods

#### Construction of the item pool

First, 110 sub-concepts of parenting styles were extracted from the two review studies that comprehensively summarized the elements of parenting styles^18,28^. Similar concepts were integrated into one category (i.e., “use of corporal punishment” and “physical punishment”), and concepts that do not refer to parenting behavior were excluded (i.e., “spousal support” and “general satisfaction”). Next, items corresponding to all sub-concepts were constructed. While creating the item pool, following points were focused on: not assessing specific parenting behavior (i.e., parenting practices) but general parenting behavior, and making the Japanese language understandable and comfortable for parents of preschool children. Three Japanese psychology researchers created the items. Two other researchers who are the parents of preschool child checked whether the items could be used for parents of preschool children and whether the Japanese language was appropriate. As a result, 82 items were formulated for assessing parenting behavior.

#### Participants and procedures

First, 20,000 adults were recruited from the monitors of Macromill Inc., one of the largest investigation companies in Japan (http://monitor.macromill.com). Next, the adults confirmed whether they had children aged 3–5 years. An online survey was conducted post obtaining their informed consent. As a result, 1236 participants completed the questionnaire on the web (50% for each gender). 59.8% of the respondents were employed, of which 12.5% were part-time workers. 97.5% of the respondents were married and the remaining 2.5% were unmarried. The participants received online payment that was plausible to be exchanged for cash or coupons as a reward for completing all the items. There were no missing values. The mean age of males was 38.483 years (*SD* = 5.393), and the mean age of females was 35.472 years (*SD* = 5.025). The median household income was 4–6 million yen, which corresponds to the middle-income group in Japan. Informed consent was obrtained from all participants. The ethical approval for conducting Study 1 and Study 2 was obtained from the ethical review board at the University of Tokyo (approval number: 22–84). All studies were conducted in accordance with the Declaration of Helsinki.

#### Materials

To assess parenting styles, participants scored on a total of 82 items on a six-point Likert scale (1 = *not at all agree* to 6 = *strongly agree*). Items were presented randomly to each respondent. Additionally, participants completed demographic items: parental gender and age, child’s gender, and family income.

#### Data analyses

All analyses in the present study were conducted using R (version 4.0.4). First, we calculated means and standard deviations for the following conditions: two categories of parental gender, three categories of parental age (20–29, 30–39, and > 40), two categories of child’s gender, three categories of child age (3, 4, and 5), and five categories of family income (< 4 million yen, 4–6 million yen, 6–8 million yen, 8–10 million yen, and > 10 million yen). Then, items were selected based on the results of the factor analyses and the analyses of a graded response model (GRM^29^) in item response theory. Regarding the factor analyses, since the meta-analysis^18^ referred to in creating the items in this study showed that parenting styles can be divided into four clusters, and since parenting styles are generally captured in four classifications^17^ as described in the theoretical background, an exploratory factor analysis (EFA) was conducted with a four-factor model. The maximum likelihood method to estimate the factor loadings, and the oblimin method were used for rotating the factor axes. Next, items with a loading of higher than 0.30 from one factor and a loading of lower than 0.30 from the other factor were selected based on the criteria used in the PSDQ development process^30^. The procedure was repeated three times until the remaining items satisfied the criteria. Finally, a confirmatory factor analysis (CFA) to examine the validity of the factor structure was performed (using the muximum likelihood method for estimation and the oblimin method for factor rotation).

In the GRM, two parameters were estimated: difficulty and discrimination^29^. The difficulty parameter refers to the extent of the ability required to rate the point. The discrimination parameter refers to the extent to which the item discriminates adequately between the respondents’ ability. In this process, the values of the discrimination parameters were focused on to select items that could discriminate between high and low abilities of the respondents with higher accuracy. The items loaded from the first factor were the items assessing positive parenting. As shown in previous studies^30^, the distributions of subscales regarding positive parenting (i.e., authoritative or warmth) tend to be skewed toward the positive side. Accordingly, those that more accurately discriminated the respondents’ ability need to be selected. Thus, the items were selected mainly based on the discrimination parameter referring to Baker’s criteria^31^. Item characteristic curves (ICC) were also examined.

### Results

First, three items showed a ceiling effect or a floor effect in conditions regarding demographic factors. Accordingly, those were removed from the item pool. Second, a factor analysis was conducted to check the factor structure of the remaining 79 items. As a result, 31 items for the first factor, 15 items for the second factor, six items for the third factor, and seven items for the fourth factor were selected. As a supplementary analysis, factor analyses with the number of factors set to five or more were conducted. In all cases, since factors with an extremely small number of items loading on the factors appeared, it was judged that these factors were not suitable as factor models for this scale. Moreover, when the four-factor structure was compared with the three-factor structure, the fit indices were slightly better for the four-factor structure (RMR = 0.040, RMSEA = 0.044, and BIC = − 10,760.451 for three-factor structure, RMR = 0.030, RMSEA = 0.040, and BIC = − 11,508.590 for four-factor structure), which suggested that the four-factor structure was more appropriate for this scale than three-factor structure.

**Figure 1:**
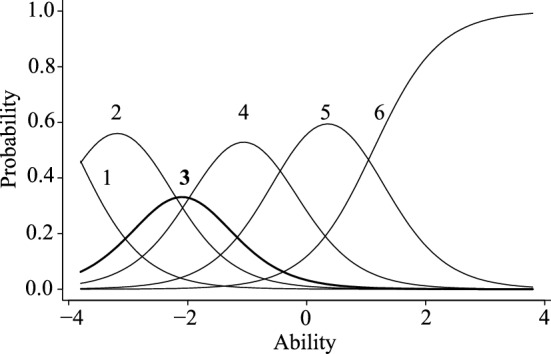
Item characteristic curves of a dropped item from the warmth sub-scale. The ability indicates the level of the respondent's characteristics that the item is measuring, and the probability indicates the probability of choosing the expected answer given that level of characteristics.

**Figure 2:**
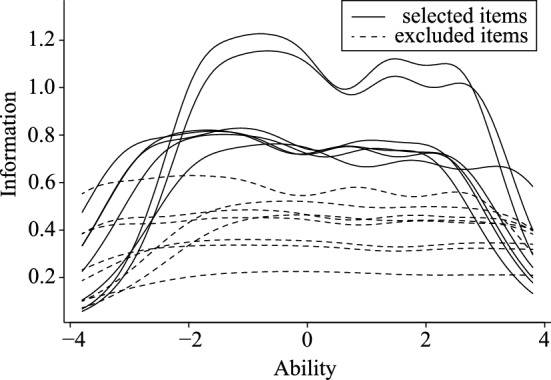
Item information curves of the original items for the hostility sub-scale.

**Figure 3:**
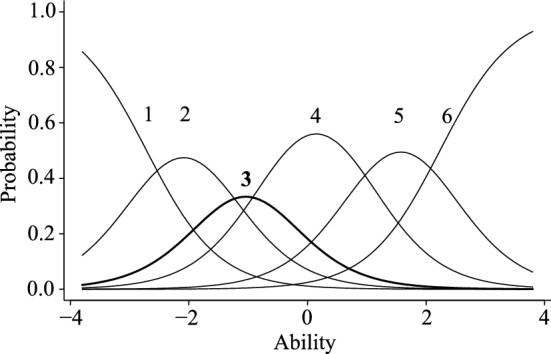
Item characteristic curves of a dropped item from the hostility sub-scale.

Because the numbers of items in the first and second factors were excessively larger than those in the third or fourth factor, some items were futher dropped from the former two factors based on the result of GRM. Regarding the 31 items of the first factor, 10 items whose discrimination parameter value was higher than 1.70 were selected based on the Baker’s criteria. In addition, as a result of the ICC of the selected 10 items, one item showed an extremely distorted ICC and was omitted (Fig. 1). Finally, nine items were selected as the first factor. Items of the second factor were also selected based on the discrimination parameter. However, only two items met Baker’s^31^ criteria. Accordingly, instead, seven of the 15 items that had richer test information than the others in Item Information Curves (IIC) were first selected (Fig. 2). Further, the ICC of the selected seven items was also examined, and one item that showed an extremely distorted ICC was removed (Fig. 3). Finally, six items were selected as the second factor. The number of items corresponding to the third and fourth factors was considered to be an adequate volume, so no additional analyses for item selection were conducted for those items. The patameters of selected items for the first and second factors are shown in the table in Supplementary information (Table S1).

Through the above procedures, a total of 28 items were selected for the present scale. Items in Japanese and English for each of the 28 items are shown in Supplementary information (Table S2). Translation from Japanese to English was performed by two bilingual researchers, and back-translation was performed to confirm the accuracy of the translation. The first factor (nine items) was named “warmth” because it consisted of items regarding positive parenting. This factor corresponds to authoritative parenting in previous studies^30^. The second factor (six items) was named “hostility,” which corresponds to the aggressive or emotional aspect of authoritarian parenting. The third factor (six items) was named “permissive parenting.” The fourth factor (seven items) was named “harsh control” which corresponds to the harshness of authoritarian parenting. As a result of the CFA, the factor model showed sufficient fit indices (CFI = 0.858, GFI = 0.987, and RMSEA = 0.056) and each item showed enough values of factor loading (Table 1). The CFAs were conducted separately for mothers and fathers. The results also showed sufficient factor structure (each item had factor loadings of 0.30 or higher from the assumed subfactor in both samples) and acceptable fit indices (CFI = 0.844, GFI = 0.987, and RMSEA = 0.057 for mothers, CFI = 0.849, GFI = 0.983, and RMSEA = 0.058 for fathers). Descriptive statistics are also shown in Table 1.

**Table 1:** Descriptive statistics of the selected items and factor loading of the CFA.

	Factor1	Factor2	Factor3	Factor4	Mean	SD	Skewness	Kurtosis
Item 1	0.702				4.541	1.026	− 0.594	0.424
Item 2	0.703				4.472	0.989	− 0.564	0.624
Item 3	0.657				4.411	0.987	− 0.323	− 0.007
Item 4	0.682				4.585	1.042	− 0.570	0.223
Item 5	0.761				4.503	1.067	− 0.681	0.390
Item 6	0.662				4.273	0.989	− 0.345	0.084
Item 7	0.628				4.204	1.006	− 0.516	0.441
Item 8	0.652				4.197	1.006	− 0.386	0.289
Item 9	0.682				4.485	1.066	− 0.552	0.155
Item 10		0.627			3.991	1.069	− 0.436	0.225
Item 11		0.675			3.387	1.214	− 0.240	− 0.415
Item 12		0.604			3.721	1.176	− 0.379	− 0.050
Item 13		0.637			4.034	1.138	− 0.511	0.291
Item 14		0.715			3.278	1.238	− 0.197	− 0.581
Item 15		0.604			3.172	1.213	− 0.127	− 0.684
Item 16			0.398		2.755	1.091	0.275	− 0.054
Item 17			0.546		3.051	1.097	0.057	− 0.333
Item 18			0.477		2.826	1.021	0.229	− 0.219
Item 19			0.662		2.888	1.099	0.130	− 0.367
Item 20			0.630		2.988	1.108	0.053	− 0.416
Item 21			0.380		2.595	1.046	0.465	0.184
Item 22				0.346	2.917	1.126	0.157	− 0.311
Item 23				0.401	3.375	1.096	− 0.168	− 0.335
Item 24				0.358	3.380	1.029	0.158	0.078
Item 25				0.403	3.615	0.948	− 0.044	0.040
Item 26				0.498	3.537	1.103	0.018	− 0.194
Item 27				0.577	4.127	0.963	− 0.435	0.604
Item 28				0.432	3.755	0.963	− 0.142	0.311
Mean	4.408	3.597	2.851	3.529				
*SD*	0.738	0.841	0.670	0.570				
Cronbach’s *α*	0.886	0.809	0.684	0.618				
*ω*_t_ (1factor model)	0.887	0.810	0.688	0.623				
SEM estimated reliability	0.887	0.810	0.690	0.617				

### Discussion

In Study 1, a new scale (i.e., the Japanese version of the parenting style scale: JPSS) with 28 original items was constructed. By focusing on the items’ characteristics, items with relatively little skewness in the distribution could be selected. In fact, the skewness values for the selected items were within normal ranges, admittedly with slight skewness (Table 1). In addition, since the number of items is half that of the PSDQ, it is possible to measure parenting styles with less burden on the participants using this scale.

The scale can then measure four aspects of parenting style with its 28 items. The four styles of the current scale basically correspond to Baumrind’s classification^15^. One of the differences from the theoretical model^15,16^ is that this scale divides the authoritarian parenting style into emotional and strict control. Considering that the sub-factors comprising the authoritarian style of the PSDQ^30^ include hostility and control parenting styles, it is reasonable that authoritarian was divided into two factors in this study. In addition, the fact that the factor analysis was conducted by relying on the data of Japanese parents’ responses may have contributed to the division. In other words, since, in general, Japanese parents have been indicated to be greater controlling than their Western counterparts^32,33^, controlling parenting styles may have been finely categorized. Accordingly, measuring two different types of controlling styles in this way provide a better precise understanding of Japanese controlling parenting. In the factor analyses, items related to neglectful parenting styles were dropped. Given an existing scale developed in Japanese^22^ also place more importance on permissiveness than neglect, the results of this study may be considered plausible.

The results of the factor analyses indicated sufficient fit indices, and the subscales also indicated sufficient internal consistency. However, the reliability coefficients of two subscales (i.e., permissive parenting and harsh control) were relatively low. Therefore, in Study 2, a number of minor adjustments were made to the sentences of a few items to measure the factor representation accurately without changing its meaning.

## Study 2

This study aimed to examine the criterion-related validity of the scale developed in Study 1 (i.e., Japanese Parenting Style Scale: JPSS). A previous study that summarized the associations between parenting styles and other variables showed that parenting style is associated not only with parental variables such as trait-level characteristics and mental health, but also with outcomes regarding child development^7^. Particularly, the present study focused on parental empathy, parental mental health, and child socio-emotional development, and the relationship between these variables and the JPSS subscales was examined. In addition, the relationship to an existing scale (the Parenting Scale^19,20^) for assessing parenting styles was examined. It was assumed that warm parenting was associated with parental empathy, low mental illness, and positive child development. In contrast, hostility parenting and harsh control were assumed to be associated with low empathy, high mental illness, and negative child development.

### Methods

#### Participants and procedures

First, a power analysis was conducted to determine the sample size based on the effect size of the correlation between parenting styles and child development outcomes in a previous study^34^, which examined the correlation in a Japanese sample. The results indicated that data needs to be collected from a sample of 593 participants to obtain the same level of effect as the previous study. Accordingly, data collection from around 600 mothers and fathers, each, for the present study was planned. As a result, it was collected from a total of 1236 participants using the same means as in Study 1. Participants who had participated in Study 1 were eleminated from the present study in advance by the investigation company. The mean age of males was 39.455 years (*SD* = 6.359), and that of females was 35.633 years (*SD* = 4.751). Most of the respondents were married (96.40%). The median household income was 4–6 million yen, which corresponds to the middle-income group in Japan. The sample had approximately the same characteristics as those in Study 1.

#### Materials

Parenting style was measured using the JPSS constructed in Study 1. The JPSS consists of four subscales: warmth (α = 0.915, ω total = 0.915), hostility (α = 0.861, ω total = 0.862), permissive (α = 0.650, ω total = 0.661), and harsh control (α = 0.662, ω total = 0.663). Each item was asked on a six-point Likert scale (1: *not true* to 6: *true*). To examine the correlations with other measures of parenting style, it was also measured using the Parenting Scale (PS)^19,20^. The translated version of the PS consists of 18 items, 10 items for over-reactivity (i.e., “I raise my voice and yell”) and 8 items for laxness (i.e., “I let my child do whatever he or she wants”). The reliability coefficients are α = 0.882, ω total = 0.882 for over-reactivity, and α = 0.733 and ω total = 0.740 for laxness. Each item asked was on a six-point Likert scale (1: *not true* to 6: *true*).

Parental empathy was measured using the Interpersonal Reactivity Index (IRI)^35,36^. The IRI consists of a total of 28 items and it measures the four aspects of individual empathetic characteristics (7 items for each of the subscales); personal distress (i.e., “When I see someone who badly needs help in an emergency, I go to pieces”), empathic concern (i.e., “I often have tender, concerned feelings for people less fortunate than me”), perspective taking (i.e., “I believe that there are two sides to every question and try to look at them both”), and fantasy scale (i.e., “After seeing a play or movie, I have felt as though I were one of the characters”). Each item was asked on a six-point Likert scale (1: *not true* to 6: *true*). The reliability coefficients are α = 0.755, ω total = 0.761 for personal distress, α = 0.709, ω total = 0.711 for empathic concern, α = 0.625, ω total = 0.646 for perspective taking, and α = 0.756, ω total = 0.764 for fantasy scale.

Parental mental health was measured using the mental health inventory (MHI)^37^. The MHI is a five-item scale assessing depression. Participants answered each item (i.e., “How much time, during the last month, have you been very nervous?”) on a six-point Likert scale ranging from 1 (*not at all*) to 6 (*always*). The reliability coefficients were α = 0.821 and ω total = 0.823.

Variables related to child development werre also measured. The Strength and Difficulty Questionnaire (SDQ^38–40^) was used for assessing the five aspects of a child’s socio-emotional development as follows: prosocial behaviors (i.e., “Considerate of other people’s feelings”), hyperactivity or inattention (i.e., “Restless, overactive, cannot stay still for long”), emotional symptoms (i.e., “Many worries or often seems worried”), conduct problems (i.e., “Often fights with other children or bullies them”), and peer problems (i.e., “Rather solitary, prefers to play alone”). The SDQ consists of 25 items, 5 for each of the subscales. The SDQ has been shown to have sufficient validity and reliability, and is used worldwide. In the present study, parents responded about their children’s development on a three-point Likert scale ranging from 0 (*not true*) to 2 (*true*). The reliability coefficients were α = 0.713, *ω* total = 0.713 for prosocial behavior, α = 0.660, ω total = 0.673 for hyperactivity or inattention, α = 0.625, ω total = 0.634 for emotional symptoms, α = 0.494, ω total = 0.533 for conduct problems, and α = 0.489, ω total = 0.494 for peer problems.

#### Data analyses

After calculating the mean and standard deviation for each variable, the Pearson correlations between variables were calculated to examine the criterion-related validity of the JSPP. Correlations were also calculated with the age and gender of the parents and their children, and household income.

### Results

The results of the correlation analyses and the mean and standard deviations are shown in Table 2. Regarding the correlations among the four subscales of the JPSS, a very weak negative correlation (*r* = − 0.061, *p* < 0.001) was found between warmth and hostility. Additionally, a slightly weak positive correlation (*r* = 0.367, *p* < 0.001) was found between hostility and harsh control. The correlations between the subscales of the JPSS and other variables are as shown in the table.

**Table 2:** Correlations between the JSPP and the other variables.

	Correlation coefficients				Descriptive statistics	
	Warmth	Hostility	Permissive	Harsh control	Mean	SD
Warmth		− 0.061*	− 0.043	0.049	4.410	0.802
Hostility			0.028	0.367***	3.644	0.910
Permissive				− 0.039	2.867	0.611
Harsh control					3.418	0.595
PS_overreactivity	− 0.321***	0.656***	0.071*	0.386***	3.241	1.023
PS_laxness	− 0.078**	0.227***	0.518***	0.050	3.478	0.766
IRI_personal distress	− 0.071*	0.342***	0.217***	0.124***	2.991	0.597
IRI_empathic concern	0.405***	− 0.018	− 0.057*	0.035	3.323	0.529
IRI_perspective taking	0.313***	− 0.162***	− 0.022	− 0.046	3.034	0.506
IRI_fantasy scale	0.182***	0.078*	0.052	− 0.034	2.982	0.647
Mental health	− 0.240***	0.302***	0.067*	0.095**	3.134	0.959
SDQ_prosocial behavior	0.331***	− 0.021	− 0.116***	0.064*	1.113	0.457
SDQ_hyperactivity/inattention	− 0.183***	0.157***	0.083**	0.097**	0.825	0.446
SDQ_emotional symptoms	− 0.183***	0.083**	0.146***	0.022	0.489	0.411
SDQ_conduct problems	− 0.304***	0.168***	0.145***	0.026	0.514	0.346
SDQ_peer problems	− 0.254***	− 0.051	0.134***	− 0.042	0.520	0.348
Age (parent)	− 0.008	− 0.224***	0.041***	− 0.024		
Gender (parent; 1 = male, 2 = female)	0.155***	0.312***	− 0.194***	0.011		
Age (child)	− 0.033	0.069*	− 0.062*	0.069*		
Gender (child; 1 = male, 2 = female)	− 0.024	− 0.018	0.035	− 0.001		
Household income	0.052	− 0.040	− 0.046	0.029		

*PS* Parenting Scale, *IRI* Interpersonal Reactivity Index, *SDQ* Strength and Difficulties Questionnaire.

***p < 0.001, **p < 0.01, *p < 0.05.

### Discussion

The aim of Study 2 was to examine the criterion-related validity of the JPSS developed in Study 1. Warm parenting showed assumed associations with all variables. In other words, there were significant associations with positive parental characteristics and positive child development, as shown in the previous rerview^7^. Hostility showed a stronger association with negative than with positive aspects of child development. The finding that hostility was associated with negative child development is supported by previous research^22^ (see also^41^). While hostility was not associated with prosociality, permissive was negatively associated with prosocially. The negative relationships between permissive parenting and child development have been shown in Japanese samples^19,34^, and the correlation coefficients are also as large as shown in such studies. The difference between hostility and permissive was also found in relation to perspective taking in the IRI scale. Hostility parenting in the JPSS is an emotional aspect of the authoritarian style. Therefore, the results are plausible in being positively associated with perspective taking, which means taking into consideration the feelings or emotions of others.

The results regarding the harsh control dimension need to be interpreted carefully. The results of the correlation analysis for the harsh control and hostility are similar, possibly because these two styles are originally from the same authoritarian style^30^. As a result, although the coefficients were slightly low, the scores on the two dimensions were significantly correlated. Nevertheless, a number of results of the correlations confirmed that the two styles were discriminable. First, hostility parenting correlated significantly with perspective taking in the IRI scale, but harsh controlling parenting did not. This result is consistent with a previous study that found perspective taking was correlated with over-reactivity and physical assault, but not with non-violent controlling discipline^42^. The second difference between hostility and harsh control is the parental gender difference. As shown in previous studies^43,44^ (see also^45^), women generally rated their hostility, trait-level anger, or assault parenting higher than men.

There also exist clear differences between the two subscales in terms of child developmental outcomes. Harsh controlling style correlated with child developmental outcome weaker than hostility, and rather, although the coefficient was very low, harsh controlling style was positively associated with child prosocial behavior. This result is consistent with some studies indicating that controlling aspects of parenting sometimes predicted positive child outcomes^46^ (see also^47^). Additionally, the results of this study reflect cultural differences. Previous studies have shown that parenting behaviors with strict discipline are less likely to lead to negative outcomes for children in the East than in the West^48^. In general, parenting styles in Asia, including Japan, are characterized by over-involvement^33,49^. Thus, high levels of controlling parenting may not be associated strongly with negative outcomes of children in this study. In summary, the results regarding harsh control are valid, considering the cultural influences.

## General discussion

The purpose of the present study was to develop a new scale for assessing parenting styles of parents with preschool children, based on a theoretical framework for a Japanese sample. Through the two studies, a new scale was constructed, and its certain validity and reliability was shown. As discussed previously, the strengths of this scale are that, unlike other Japanese scales, this scale was designed for parents with preschool children and was developed based on the theoretical background.

However, there are a few limitations to this scale. For example, in the present study, some items were removed based on the discriminative value of the GRM. This allowed us to develop a scale with a small number of items and still with high consistency within the subscales, which is one of the aforementioned strengths of this scale. However, there is a trade-off between the number of items and content validity, thus reducing the number of items may have narrowed the range of what could be measured with this scale. In this study, the content of the items was checked by all the authors many times, and it was confirmed that the current items can measure the concepts as assumed, but perhaps the content validity should have been examined more rigorously, for example, by using a content validity index^50^.

In this study, only cross-sectional correlations were examined. Particularly, parenting styles are considered as a determinant of child development^7^. Therefore, the causal effects of parenting styles on child development need to examined in future studies for further validity confirmation. There is also a limitation regarding the measurement methods in Study 2 since only self-reported measures were relied on. Therefore, parenting behavior and child developmental outcomes need to be assessed by observing and examining its association with the JPSS. Another limitation is the low reliability of the subscales of the SDQ. The results may have led to overestimation or underestimation of the correlation between the JPSS and the SDQ.

Despite these limitations, the JPSS is a scale with adequate reliability and validity to measure the parenting styles of Japanese parents who have preschool children. Since this scale has a small number of items compared to the existing scales^19,22^, it can be used relatively easily in practice and research. With the development of this scale, it is expected that the effects of parenting on preschool children’s development can be examined in more detail than is currently thought possible. Further, it is also expected that this scale will lead to progress in the international comparison research on parenting styles.

## Data availibility

The data of the current study are available from the corresponding author on reasonable request.

## Supplementary Information

Supplementary Tables.
