# Supplementary material for: Development of the Japanese Parenting Style Scale and examination of its validity and reliability
Source: Sci Rep. 2022 Oct 27;12:18099. doi: 10.1038/s41598-022-23153-5 (PMC9613974; doi:10.1038/s41598-022-23153-5)
Supplement: Supplementary file 1 — Supplementary Tables. [file 41598_2022_23153_MOESM1_ESM.docx]

**Supplementary information**

**Development of the Japanese Parenting Style Scale and examination of its validity and reliability**

Keisuke Okubo^1*^, Yinqi Tang^2^, Jiwon Lee^3^, Toshihiko Endo^1^, and Sachiko Nozawa^1^

^1^ The Center for Early Childhood Development, Education, and Policy Research, The University of Tokyo, Japan

^2^ Kyoai Gakuen University, Japan

^3^ Benesse Educational Research and Development Institute, Tokyo, Japan

*Corresponding author: Keisuke Okubo. E-mail: kokubo@p.u-tokyo.ac.jp

| Items for Factor 1 | | | | | | | |
| --- | --- | --- | --- | --- | --- | --- | --- |
|  | a |  | b_1_ | b_2_ | b_3_ | b_4_ | b_5_ |
| Item 1 | 1.911 |  | -3.286 | -2.415 | -1.461 | -0.097 | 1.321 |
| Item 2 | 2.109 |  | -3.229 | -2.378 | -1.405 | 0.024 | 1.465 |
| Item 3 | 1.747 |  | -3.788 | -2.596 | -1.311 | 0.164 | 1.637 |
| Item 4 | 1.774 |  | -3.544 | -2.558 | -1.503 | -0.130 | 1.237 |
| Item 5 | 2.067 |  | -3.102 | -2.200 | -1.269 | -0.091 | 1.346 |
| Item 6 | 1.803 |  | -3.710 | -2.348 | -1.163 | 0.332 | 1.869 |
| Item 7 | 1.741 |  | -3.268 | -2.228 | -1.090 | 0.411 | 2.077 |
| Item 8 | 1.717 |  | -3.343 | -2.312 | -1.076 | 0.442 | 2.012 |
| Item 9 | 1.763 |  | -3.404 | -2.362 | -1.343 | -0.046 | 1.390 |
| Items for Factor 2 | | | | | | | |
|  | a |  | b_1_ | b_2_ | b_3_ | b_4_ | b_5_ |
| Item 10 | 1.641 |  | -3.047 | -1.872 | -0.888 | 0.702 | 2.313 |
| Item 11 | 2.006 |  | -1.778 | -0.912 | -0.125 | 1.360 | 2.582 |
| Item 12 | 1.616 |  | -2.408 | -1.467 | -0.509 | 1.052 | 2.463 |
| Item 13 | 1.628 |  | -2.719 | -1.797 | -0.951 | 0.597 | 2.024 |
| Item 14 | 1.943 |  | -1.662 | -0.791 | 0.000 | 1.437 | 2.776 |
| Item 15 | 1.575 |  | -1.850 | -0.754 | 0.139 | 1.773 | 3.438 |

**Table S1**. The estimated parameters in the analyses of the GRM for items of Factor 1 and Factor2. Note. a = item discrimination, b*n* = latent ability of respondents to have a 50% probability to rate the nth and *n+1*th response categories.

| 1 | I try to play with my child when I have time. |
| --- | --- |
|  | 時間があるときは，子どもと一緒に遊ぶようにしている。 |
| 2 | I actively talk to my child. |
|  | 子どもに対して，自分から積極的に話をする。 |
| 3 | I’m willing to have a physical contact with my child. |
|  | 子どもとスキンシップを進んでとっている。 |
| 4 | I interact with my child in a way that makes it easy for him/her to talk to me about anything. |
|  | 子どもが自分に何でも話してくれるように接している。 |
| 5 | Even when my child is crying, I stay calm and care what my child is thinking. |
|  | 子どもが泣いているときでも，落ち着いて，子どもが何を思っているかを気にかける。 |
| 6 | When my child is crying or happy, I am there for him/her with the same feelings. |
|  | 子どもが泣いたり喜んだりしているときは，同じ気持ちになって寄り添う。 |
| 7 | I try to spend as much time as possible with my child. |
|  | なるべく，子どもと一緒に過ごす時間を十分にとっている。 |
| 8 | When my child is depressed, I am there for him/her as much as I can. |
|  | 子どもが落ち込んでいるときは、できる限りそばにいる。 |
| 9 | When I have time, I will try to be with my child as much as possible. |
|  | 時間があるときは，なるべく子どものそばにいるようにする。 |
| 10 | I often get angry at my child for what he/she says and does. |
|  | 子どもの言動に対して，怒ってしまうことがよくある。 |
| 11 | I sometimes say things to my child in a commanding tone of voice. |
|  | 子どもに，つい命令口調で物事を伝えることがある。 |
| 12 | I sometimes get irritated by my child’s behavior. |
|  | 子どもの振る舞いにイライラすることがある。 |
| 13 | When I get angry with my child, I sometimes say mean things on purpose. |
|  | 子どもに腹が立ったときに，わざと意地悪なことを言うことがある。 |
| 14 | I sometimes vent my stress and anger on my child. |
|  | 子どもに自分のストレスや怒りをぶつけてしまうことがある。 |
| 15 | I sometimes get frustrated with my child and interact him/her aggressively |
|  | 子どもにイライラして，攻撃的に接することがある。 |

**Table S2**. Items of the JSPP in English and Japanese.

| 16 | I sometimes give special treatment to my child when he/she stays with other children. |
| --- | --- |
|  | 他の子どもと一緒にいるときに，自分の子どもを特別扱いすることがある。 |
| 17 | Even when my child causes the same trouble, I sometimes get angry and sometimes not. |
|  | 子どもが同じ問題を起こしても，怒ったり怒らなかったりすることがある。 |
| 18 | When my child is working on something, I sometimes find myself helping him/her. |
|  | 子どもが何かに取り組んでいるとき，つい手助けすることがある。 |
| 19 | When my child doesn’t listen to me, I give up and do what he/she asked. |
|  | 子どもが言うことを聞かないときは，諦めて子どもの言う通りにする。 |
| 20 | When my child cry or get angry, I am at his/her mercy. |
|  | 子どもが泣いたり怒ったりしたら，子どもの言うことを聞いてしまう。 |
| 21 | I forgive my child for doing something wrong without getting angry. |
|  | 子どもが何か間違ったことをしても，怒ることなく許す。 |
| 22 | When my child makes a mistake, I try to make him/her think, “It’s my fault.” |
|  | 子どもが失敗したときは，「私/僕が悪いんだ」と子どもに思わせるようにしかる。 |
| 23 | When my child is crying, I tell him/her to stop crying quickly. |
|  | 子どもが泣いているときは，早く泣き止むよう言い聞かせる。 |
| 24 | I discipline my child thoroughly to the details. |
|  | 子どものしつけは細かいところまで行き届くように徹底している。 |
| 25 | Even when my child fails, I won’t spoil my child. |
|  | 子どもが失敗しても、甘やかすようなことは言わない。 |
| 26 | I will scold severely regardless of the reason if my child breaks a rule. |
|  | 子どもがルールを破ったら，理由に関わらず，厳しくしかる。 |
| 27 | When my child breaks a rule, I remind him/her over and over again to obey it next time. |
|  | 子どもが決まりを破ったときは，次からは守るように何度も言い聞かせる。 |
| 28 | If my child can’t do something, I make him/her try to do it again and again until he/she can do it. |
|  | 子どもにできないことがあったら，できるようになるまで何度もやらせている。 |

**Table S2.** *continue*
